# Progress of the Surgery of the Vermiform Appendix

**Published:** 1902-07-05

**Authors:** 


### PROGRESS OF THE SURGERY OF THE VERMIFORM APPENDIX.

**Appendicitis.**—From a study of the pathology of appendicitis Stinson[1] draws the following conclusions :—1. That, as appendicitis is strictly a surgical disease, the earlier it is operated upon the better for the patient. 2. That cases operated upon early should have no mortality. 3. That during all appendicitis operations the appendix should be removed, provided irreparable damage is not done in attempting to find or remove it. 4. That where there is a local or general infection the abscess cavity or cavities should be freely opened, all adhesions separated, all pus, shreds, etc., cleaned out, all inflamed or pathologic omentum excised, and all pathologic intestines and infected portions of abdomen be freely irrigated with hot water or hot saline solution till the fluid comes away clear ; the intestines, etc., are then dried with sponges and returned to their normal positions. 5. That fæcal concretions are more apt to be present as exciting causes of appendicitis than foreign bodies. 6. That fæcal concretions closely resemble some foreign body of light weight, *i.e.* grape seeds, cherry stones, etc., and that when one is in doubt whether the material is a concretion or foreign body it should be carefully examined microscopically and chemically to determine the exact characters. Cotterill[2] is in favour of operating after recovery from a first attack of appendicitis for the following reasons :—1. A large percentage of all cases, estimated by various writers at from 25 to 40 per cent., had second attacks. In all but the mildest cases a second attack is the usual rule. 2. No one can predict that such a second attack will not be perforative nor prove fatal, in spite of treatment. Operative treatment during the acute attack is, of course, a much more serious undertaking than when done in an interval. 3. Every attack adds to the extent and strength of the adhesions, and thus increases the difficulties and dangers of the operation, sometimes to a very great degree. 4. The mortality of the operation when done in an interval, especially after a first attack when the adhesions are at a minimum, is comparatively trifling, say 1 to 2 per cent. at the outside, in the hands of competent surgeons. Ochsner,[3] writing of the differential diagnosis of acute appendicitis, says it is most commonly mistaken for the following conditions, in the order named :—1. Gastritis or gastric ulcer. 2. Enteritis. 3. Gall-stone colic. 4. Peritonitis, due to infection through the Fallopian tubes in women. 5. Extra-uterine pregnancy. 6. Renal colic. 7. Perforative intestinal ulcer due to typhoid fever, tuberculosis, actinomycosis, or carcinoma. 8. Intussusception. 9. Strangulated hernia. 10. Intestinal obstruction due to bands of adhesions. Barling[4] draws attention to the low mortality of suppurative appendicitis if the removal of the appendix is not insisted upon. In 74 cases there were two deaths. The appendix was only removed 25 times, and in 49 it was not removed. Battle[5] considers that in suppurative appendicitis in which the appendix has not been removed that as soon as possible after the healing of the abscess appendicectomy is desirable. Berry[6] says when the appendix is always removed the death rate is high. There are two schools of surgery—the one favouring a small, the other a large incision. Of 41 cases with a small incision, there were only 2 deaths ; whereas in 39 cases treated by various surgeons without regard to the adhesions there were 32 deaths. Berry and his colleagues at the Royal Free Hospital prefer to wait for adhesions to form before operating ; and out of 53 cases had had 49 recoveries and 4 deaths.

Da Costa[7] has examined the blood of 118 cases of appendicitis treated by operation, and draws the following conclusions :—1. The average case of appendicitis before operation shows a loss of about 30 per cent. of hæmoglobin, and of more than half a million erythrocytes per cubic millimetre. Occasionally the anæmia is of a grade so high that it appears to constitute in itself a serious complication, and to raise a doubt as to the safety of surgical interference, should the latter otherwise be indicated. Doubts on this score, however, have not been justified by the records of the cases included in this series. 2. Moderate leucocytosis may occur, both in the absence and in the presence of an abscess and its consequences. It accompanies about 35 per cent. of non-purulent and 90 per cent. of purulent cases. 3. Leucocyte counts, ranging between 10,000 and 15,000 or 17,000, cannot be depended upon to reflect the nature of the local lesion, since this degree of increase may be found both in mild catarrhal and in purulent cases. Counts of 20,000 or more almost invariably indicate the presence of pus, gangrene, or general peritonitis, one or all. 4. Leucocytosis may be absent both in trivial catarrhal and in fulminant cases, as well as in forms of circumscribed abscess. 5. In operative cases thorough evacuation of the abscess is followed within a few days by a decline to normal in the number of leucocytes, provided that the recovery of the patient is uneventful. Persistence of a leucocytosis after the third or fourth day following the operation may usually be attributed either to undrained pus pockets, to general peritonitis, or to both of these factors. Making a "conservative estimate," he finds that in one out of four cases of appendicular abscess the diagnosis is justified by the leucocyte count. As a means of diagnosing appendicitis from the conditions most closely resembling it—ovarian abscess, pyosalpinx, ectopic pregnancy, perinephric abscess, hepatic abscess, and malignant disease of the cæcum—examination of the blood is useless, for all the conditions just named give rise to similar blood changes. But if the diagnosis lie between appendicitis and typhoid fever, the former is suggested by the presence of leucocytosis, which occurs in the latter only when perforation takes place. Leucocytosis also excludes such non-inflammatory conditions as simple enteralgia, lead colic, ovarian neuralgia, ovarian cyst, and movable kidney.

Ochsner[8] considers that diffuse peritonitis complicating appendicitis is produced by the peristaltic motion of the small intestine, carrying the infection from the perforated or gangrenous appendix to the other portions of the peritoneum and changing a circumscribed into a general peritonitis. This can be prevented by prohibiting the use of every kind of food and cathartics by the mouth, and by employing gastric lavage in every case in which there are remnants of food in the stomach or in the intestines above the ileo-cæcal valve, as indicated by the presence of nausea, or vomiting, or meteorism. The patient can be supported by nutrient enemata. This form of treatment, when instituted early, will change the most violent and dangerous form of acute perforative or gangrenous appendicitis into a comparatively mild and harmless form. The patient should be permitted to recover fully from his acute attack before an operation is performed, except in cases encountered within the first thirty-six hours after the beginning of an attack, or in case of the formation of a superficial circumscribed abscess. Thomas[9] records a case of parotitis following an operation for appendicitis; Wallace[10] a case in which the appendix vermiformis was passed in stool after several attacks of appendicitis; and Galton[11] a case of appendicitis in which the appendix was lodged in, and adherent to, the femoral canal.

[1] Med. Rec., March 22, 1902, p. 444. [2] Scottish Med. and Surg. Journ., Sept. 1901, p. 202. [3] Med. Rec., Aug. 17, 1901, p. 280. [4] Brit. Med. Journ., Feb. 22, 1902, p. 455. [5] Lancet, Jan. 18, 1902, p. 144. [6] Brit. Med. Journ., Feb. 22, 1902, p. 455. [7] Annals of Surgery, Nov. 1901, p. 645; and Lancet, Jan. 4, 1902, p. 45. [8] Amer. Journ. Med. Sci., Dec. 1901, p. 879. [9] Lancet, Dec. 28, 1901, p. 1796. [10] Med. Rec., Nov. 16, 1901, p. 786. [11] Lancet, March 29, 1902, p. 888.

